# An Edge-Computing-Based Integrated Framework for Network Traffic Analysis and Intrusion Detection to Enhance Cyber–Physical System Security in Industrial IoT

**DOI:** 10.3390/s25082395

**Published:** 2025-04-10

**Authors:** Tamara Zhukabayeva, Zulfiqar Ahmad, Aigul Adamova, Nurdaulet Karabayev, Assel Abdildayeva

**Affiliations:** 1 Department of Information Systems, L.N. Gumilyov Eurasian National University, Astana 010000, Kazakhstan; zhukabayeva_tk@enu.kz (T.Z.); aigul.adamova@astanait.edu.kz (A.A.); assel.abdildaeva@kaznu.edu.kz (A.A.); 2 Department of Computer Engineering, Astana IT University, Astana 010000, Kazakhstan; 3 Department of Computer Science and Information Technology, Hazara University, Mansehra 21300, Pakistan; 4 Department of Artificial Intelligence and Big Data, Al-Farabi Kazakh National University, Almaty 050040, Kazakhstan

**Keywords:** network analysis, clustering, cybersecurity, intrusion detection and prevention, machine learning, cyber–physical systems, industrial IoT

## Abstract

Industrial Internet of things (IIoT) environments need to implement reliable security measures because of the growth in network traffic and overall connectivity. Accordingly, this work provides the architecture of network traffic analysis and the detection of intrusions in a network with the help of edge computing and using machine-learning methods. The study uses k-means and DBSCAN techniques to examine the flow of traffic in a network and to discover several groups of behavior and possible anomalies. An assessment of the two clustering methods shows that K-means achieves a silhouette score of 0.612, while DBSCAN achieves 0.473. For intrusion detection, k-nearest neighbors (KNN), random forest (RF), and logistic regression (LR) were used and evaluated. The analysis revealed that both KNN and RF yielded seamless results in terms of precision, recall, and F1 score, close to the maximum possible value of 1.00, as demonstrated by both ROC and precision–recall curves. Accuracy matrices show that RF had better precision and recall for both benign and attacks, while KNN and LR had good detection with slight fluctuations. With the integration of edge computing, the framework is improved by real-time data processing, which means a lower latency of the security system. This work enriches the knowledge of the IIOT by offering a detailed solution to the issue of cybersecurity in IoT systems, based on well-grounded performance assessments and the right implementation of current technologies. The results thus support the effectiveness of the proposed framework to improve security and provide tangible improvements over current approaches by identifying potential threats within a network.

## 1. Introduction

The Industrial Internet of things (IIoT) is a revolution in industrial systems that includes sensor technologies, data, and connectivity [1,2]. The IIoT uses a network of connected intelligent devices and systems that work in real time to enhance industrial processes. Integrating sensors and actuators into the machinery and equipment of an industry, IIoT systems gather large volumes of data on the machinery performance, the surrounding environment, and industry parameters [3,4,5]. This information is then converted into value-added outcomes by employing complex analysis and learning models to help decision-makers and increase organizational effectiveness. The basic advantages of the IIoT are mostly reflected in the concepts of automation, maintenance prediction, and operation insight. In a similar way, the IIoT helps organizations to use real-time monitoring and real-time analytics for anticipative maintenance approaches that can predict the failure of the used equipment before such a failure happens, which, in turn, reduces both the time that the equipment is not in use and the amount of money spent on maintenance [6]. Moreover, the IIoT has made operational visibility possible and gives an all-round view of the operations, making it easier to decide what next step should be taken. Consequently, the IIoT leads to critical advancements in the efficiency, protection, and utilization of resources in many industries, such as manufacturing, energy, transport, and farming industries. The IIoT is the integration of different smart devices and data analysis to redesign the future of industrial processes and create a brand-new generation of more effective and more robust industrial environments [6,7,8].

The security of cyber–physical systems (CPSs) in the IIoT is an important issue because it might become widely integrated into various industrial processes soon [9,10,11]. CPSs in IIoT applications interlink tangible equipment as well as mechanisms with intangible systems, which facilitate information sharing and command execution [12]. But this integration also presents huge security issues that need to be solved to counter various threats and risks [13,14]. The primary security concern is unauthorized access and control, as it creates an obvious information security threat. Several risk factors that affect the IIoT deployments include hacking and malware; they result in the potential compromise of the IIoT system due to the susceptibility to interferences, interruptions, or even damaging impacts on machinery data. Strengthening and enhancing authentication and authorization frameworks remains critical in safeguarding industrial systems from cyber threats [15,16,17,18]. Another important challenge is the issue of data integrity and data security. IIoT systems collect a lot of data; they include operational data, and maintenance data, as well as control data [18,19,20]. Such information must be appropriately transferred and archived in a manner that would not allow third-party intrusion. The use of ensembles and safe channels is necessary when it comes to protecting data from cybercriminals [7]. The nature and variety of IIoT systems and applications make it difficult to achieve coherent security requirements and policies. Having this many gadgets, protocols, and software platforms makes the IIoT systems heterogeneous and, hence, makes it challenging to implement uniform security measures. Measures such as system updates, vulnerability management, and IDS implementation should be taken to address these challenges. Protection against cyberattacks is of great importance concerning the sustainability and availability of IIoT systems and networks. Every business is prone to security incidents, and strong incident response protocols and disaster management strategies reduce the consequences and disruption of the industrial processes should they be attacked [21,22].

Edge computing is an important part of the IIoT since it optimizes the performance, dependability, and quickness of industrial processes [9,23]. It involves working with information nearer to where it is created; for instance, in the sensors and machines instead of going to massive cloud information hubs [24,25]. IIoT ecosystems produce huge amounts of data from sensors and other devices in the system. Relaying all this information to a central cloud may cause network bandwidth issues and, subsequently, high latency. Edge computing relieves this problem by processing and filtering a lot of data at the edge without sending raw data to the cloud. This maximizes the utilization of bandwidth and puts less strain on the supporting structures in the network. Edge computing enables the expansion of IIoT systems by the incorporation of more edge devices and sensors at the edge without overloading the central cloud. Such decentralization also opens more opportunities for more efficient and adaptable industrial setups, as more processing and storage resources can be deployed at different locations [9,26,27,28].

Security is one of the main issues of the IIoT, where the protection of cyber–physical systems becomes challenging due to the complexity and connectivity [29]. Since IIoT systems pull together increased numbers of sensors, devices, and communication networks, the exposure to cyber threats also increases, and, thus, it calls for effective security. The more complex these networks become and the faster they grow, the harder it is for traditional security solutions to protect these systems, which is why new approaches are needed in this sector. The advanced usage of edge computing can provide a solution to these security issues. Edge computing means that the data processing is performed nearer to the source, which involves IoT devices and sensors, unlike depending on a centralized cloud platform [30]. This approach not only saves time and bandwidth but also increases the ability to analyze information and make decisions in real time. If edge computing nodes are integrated into the IIoT network, traffic can be analyzed, and, potentially, threats are detected in real time, which makes the response time faster [31,32,33]. Another aspect addressed in improving security in IIoT systems is based on including extended traffic analysis and intrusion detection methods [2]. Clustering traffic data can be applied to analyze normal and even detect abnormal traffic flow by collecting similar datasets, which is beneficial for filtering out legitimate and illegitimate traffic. When coupled with IDSs such as k-nearest neighbors (KNN) [34,35,36], random forest (RF) [34,37,38], and logistic regression (LR) [34,36,39,40], potential intrusions can be identified and classified correctly using the observed traffic pattern.

This research is driven by the growing importance and critical nature of cybersecurity in industrial IoT systems. With growing numbers of industries implementing IoT into their processes, the intensity and variety of network load remain high, which poses enormous problems for the creation of effective security systems. Industrial IoT systems are embedded in many industries to facilitate the control and monitoring of critical processes in manufacturing and energy, among other industries in transportation. In such environments, the potential of cyber threats is vast, thus implying that, even though protective measures are important, they are sensitive. Traditional approaches often struggle to adapt to the diverse and evolving traffic patterns in industrial IoT networks, and this makes them less effective against sophisticated cyberattack strategies [13,16,41,42,43,44]. Moreover, this work is inspired by the desire to move beyond traditional security solutions and come up with better, more flexible approaches to network analysis and intrusion detection.

The main contributions of the proposed research study are highlighted below:

- We propose a comprehensive framework that integrates network traffic analysis, clustering, intrusion detection, and edge computing to address the complexity and security challenges of cyber–physical system security in industrial IoT.
- We employ k-means and DBSCAN clustering methods to effectively segment network traffic and, therefore, detect different traffic scenarios in IIoT networks as well as potential discrepancies.
- We implement and compare three machine-learning models (KNN, RF, and LR) to detect intrusions and found the models performing well with a high accuracy to differentiate between benign and malicious traffic.
- We provide the practical mechanism of incorporating edge computing within the proposed framework.
- We provide recommendations regarding clustering and machine-learning approaches that are suitable for practical implementation in actual industrial IoT environments.

We organized the remaining part of the paper as follows: Section 2 describes the related work. Section 3 employs the system design and model. Section 4 presents a performance evaluation, and, finally, Section 5 concludes the article with several future directions.

## 2. Related Work

We reviewed the related work in context with security issues in cyber–physical systems, edge computing integration, network analysis, and intrusion detection methods for cyber–physical systems.

With the emergence of cyber–physical systems, people confronted new opportunities. Protecting information from cyber–physical systems is one of the most challenging questions in a vast number of protections against cyber threats. The purpose of the study presented in [16] is to review the literature related to the security of cyber–physical systems and categorize them. The nature of cyber–physical systems is briefly described in terms of philosophical problems. The authors also identified the main types of attacks and threats against cyber–physical. In the recent past, the research on the CPS has been receiving much attention in the research community as well as in industries [27]. The first challenge to the rapid deployment of CPS applications is the lack of an idea on how best to deal with the large volumes of data that the applications generate for decision-making. In response to this challenge, scholars propose the concept of incorporating edge computing, or edge–cloud computing, into the design of CPSs. However, this coupling process brings a diversity of issues in the QoS of CPS applications into question. In [27], the authors offer an overview of the edge-computing- or edge–cloud-computing-assisted CPS designs from the QoS optimization point of view. The authors provide a brief of the state-of-the art works addressing divergent issues related to QoS optimization.

The transformation of the fourth industrial revolution uses a CPS that protects Supply Chain 4.0. It combines manufacturing information with Internet communication technology to design a smart CPS that captures product data from manufacture to customer delivery by the Internet of things. The research presented in [13] employs a ML method for network anomaly detection and building models to identify DDoS attacks targeting Industry 4.0 CPSs. The limitations of the previous techniques, artificial data, and small datasets are eliminated by collecting real-world network traffic data from a semiconductor production factory. The presented PCA-BSO algorithm is used to identify the most significant features by using their eigenvalues, though the feature that has the largest eigenvalues is not necessarily beneficial to improving the classification. As for the supervised ML algorithms, the simulations are performed to compare the results of the algorithms. Hackers consistently create new approaches on how to confuse and deceive victims, making cybersecurity an ongoing process aimed at maintaining the availability, confidentiality, and integrity of computer systems [41]. Proactive protection is engaged by employing machine learning (ML) as an effective approach to the intelligent cyber analysis of recurring patterns of successful attacks. However, two significant drawbacks hinder the widespread adoption of ML in security analysis: relatively high computing overheads and the requirement of specialized frameworks. The study presented in [41] is intended to establish the numerical value by which a hub can improve the safety of an ecosystem. Traditional cyberattacks were performed on an IoT network in a smart house to test the functionality of the hub. The robustness of the IDS against adversarial machine-learning (AML) attacks was explored, in which models are attacked with adversarial samples to exploit vulnerabilities.

With advanced technology, most devices have been created to share extensive information and work in unison as Edge Intelligence in Smart Cities (EISC). In circumstances where personal data are processed, great care must be taken to make sure that personal data are not disclosed, and there should not be any disclosure of any information. Systems such as IDSs, are needed to penetrate firewalls, anti-virus programs, and other protective equipment to ensure total system protection in smart operating systems. There are three aspects to an IDS: the intrusion detection method, the architecture, and the intrusion response method. In [42], linear correlation feature selection methods and cross-information are used in combination. The dataset used in the article is KDD99. The paper, therefore, discusses using two approaches in attack prediction in intrusion detection systems known as INTERACT and a multilayer perceptron (MLP). Since the record count for each attack type may not be the same, one of the recommendations is to keep on using data-balancing approaches.

A comparison of the proposed framework with previous studies is shown in Table 1, which highlights the ML techniques used, the features considered, and the datasets utilized. The traditional approaches struggle to adapt to the diverse and evolving traffic patterns in industrial IoT networks and are less effective against advanced cyberattack strategies as compared to the proposed approach.

## 3. System Design and Model

**Figure 1:**
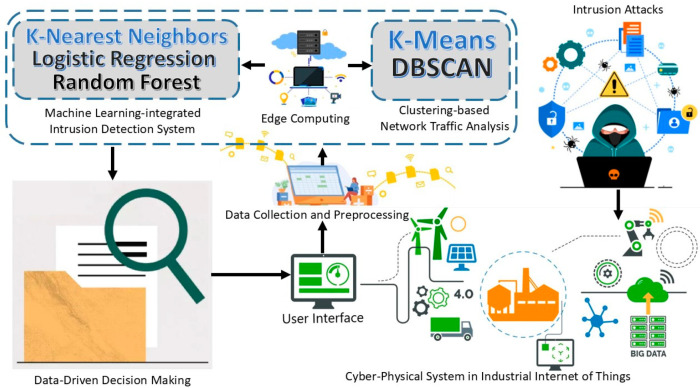
An edge-computing-based integrated framework for network traffic analysis and intrusion detection to improve CPS security in industrial IoT.

We propose an edge-computing-based integrated framework for network traffic analysis and intrusion detection to improve CPS security in industrial IoT, as illustrated in Figure 1. The framework is intended for use in the IIoT for CPSs, where traffic is constantly analyzed for security threats. The data used in the study are collected from a CPS within the IoT environment; the CPS collects different types of network traffic data. These traffic data also consist of normal and intrusion instances. Raw network traffic undergoes preprocessing before analysis. Preprocessing encompasses data cleansing, and scaling, as well as data transformation and dimensionality reduction.

Two clustering algorithms, namely, k-means and DBSCAN, are used to analyze the network traffic data in an unsupervised manner. The purpose is to find specific groups of points that characterize other traffic behaviors or the presence of outliers, which can signal cyberattacks. The algorithm, k-means clustering, divides the data into predetermined clusters, implying well-developed clusters. Density-based spatial clustering of applications with noise (DBSCAN) finds clusters and noise with different densities in clusters. The preprocessed data are also used in evaluating the performance of three supervised machine-learning models for intrusion detection. The basic and efficient KNN algorithm in this study has allowed us to gain good performance in terms of precision, recall, and F1 scores. The better precision and recall of the RF ensemble-learning method were also achieved when used solely for distinguishing both benign and attack traffic, particularly in several attack classes. LR has the ability to show good detection performance in intrusion detection, and, therefore, it was also used in the proposed framework. An important part of the given framework is the incorporation of edge computing. Since the analysis of the network traffic data is performed at the network periphery, the proposed framework prevents the high latency that is inherent to most existing solutions, as well as enabling real-time intrusion detection. This has the effect of improving the system’s capability to rapidly identify antecedents and respond to dangerous elements. This is followed by the network traffic analysis and intrusion detection, after which the system uses a data-based decision-making function to decide on the best course of action to be taken in case of any security threat. In the occurrence of recognized anomalies or intrusions, the system can perform such actions as alerting security staff, disconnecting the infected devices, or automatically starting defense procedures.

The elevation of security measures in the cyber–physical system of the IIoT is confirmed to be effectively addressed by the proposed framework. By applying clustering for network data analysis and machine learning for intrusion detection with edge computing for real-time operation, the proposed approach guarantees the swift and precise detection of security threats. The system helps improve cybersecurity by providing the capability for a faster response and better decision-making to protect the infrastructure from industrial IoT.

### 3.1. Implementation of Edge Computing in the Proposed Framework

**Figure 2:**
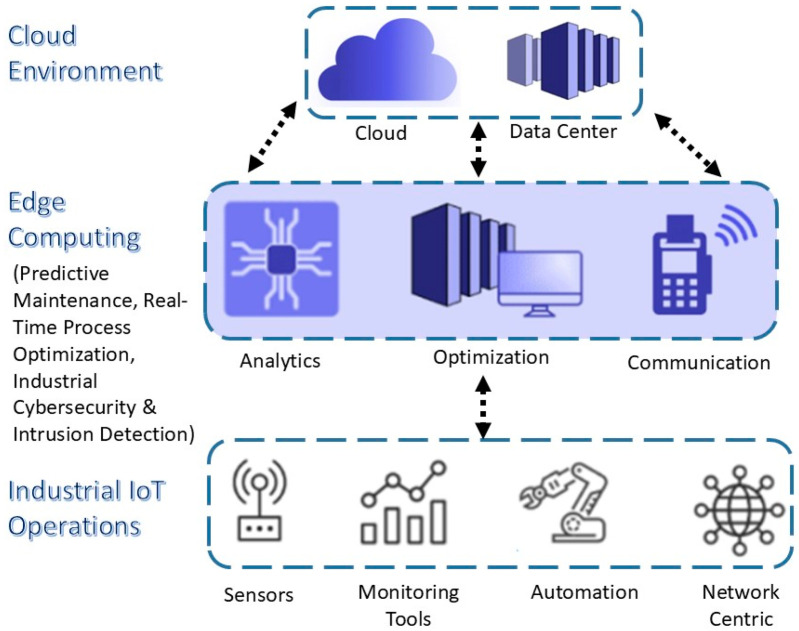
Edge computing implementation in IIoT.

By integrating edge computing in the proposed framework, it enhances the performance on network traffic analysis and intrusion detection by the timely processing of the data at the edge of the network. As shown in Figure 2, the distributed task computing is carried out at the edge layer instead of depending on centralized cloud servers alone, which distributes the computation over multiple edge devices, including routers, gateways, and fog nodes. There are these intermediate processing units, which are edge nodes, which analyze the network traffic closer to the data source and, thus, reduce the latency and response time for security threats in the IIoT environments. The framework uses edge computing to make sure that critical security operations such as clustering and machine-learning-based intrusion detection as well as an automated threat response are carried out efficiently without burdening the central cloud infrastructure.

Real-time data collection and preprocessing at the edge is the first step towards the implementation of edge computing. As IIoT environments have lots of CPSs, the network traffic generated from these CPSs is large and includes normal and anomalous data packets. All network traffic would be routed to a cloud-based security system for analysis. In the proposed framework, however, data are captured and preprocessed at edge-computing devices, and only the relevant data are sent to the cloud. The first part of edge computing is data cleansing, feature extraction, normalization, and dimensionality reduction, which are carried out as preprocessing tasks at the edge. These operations discard redundant or noisy data and only pass on useful and security-relevant data for further processing. The reduction in network congestion and maximization of bandwidth utilization are significantly achieved as the amount of raw data transmitted to central servers is minimized.

With the data preprocessed, it is fed at the edge and analyzed using clustering techniques such as k-means and DBSCAN. The clustering algorithms are executed on the edge nodes to segment network traffic into different behavioral patterns for the purpose of the early detection of anomalies that can be indicative of potential cyber threats. K-means clustering is highly effective for clustering structured traffic patterns so that normal and suspicious activities can be identified with high precision. However, DBSCAN is useful for detecting irregular, sparsely distributed, and noisy anomalies and, thus, is very useful in detecting outliers and noise in network traffic. By performing clustering at the edge, the system can quickly identify potential security breaches and forward only the high-risk data instances to the central security server for further investigation. On top of this, the framework deploys machine-learning intrusion detection models at the edge for security-monitoring enhancement. In order to classify network traffic in real time, we implement three machine-learning algorithms on edge-computing devices using KNN, RF, and LR. The models that have been trained on historical intrusion datasets analyze the incoming network packets and decide if they are normal or attack traffic. For its initial classification step, the KNN algorithm is used as an algorithm with a simple implementation and high efficiency, while the second has the higher accuracy to classify whether the traffic is benign or malicious—an RF model, which has the robustness for distinguishing between benign and malicious traffic. The LR model helps in further improving the classification capability by modeling the probability of the occurrence of the attack. Given that early detection and mitigation are crucial, these computations can be carried out at the edge on time compared to cloud-based solutions, thereby drastically reducing the time taken for detection.

The important advantage that is brought in by the incorporation of edge computing in the proposed framework is that it helps in providing real-time decision-making as well as automated response mechanisms. In the case of an intrusion or anomaly at the edge, the system responds immediately and without waiting for any orders sent by the centralized cloud server. Actions that may be implemented by the response part of these are isolating compromised devices, blocking suspicious network traffic, updating firewall rules dynamically, or sending the alerts to the security personnel. Edge computing is decentralized, which means security incidents are dealt with at the place of origin, thereby stopping any potential threats from spreading throughout the whole IIoT network. Edge computing makes it possible to have the continuous monitoring of the network behavior and then respond with adaptive security mechanisms on the fly according to the changing nature of the cyber threats.

### 3.2. Algorithm for Edge-Computing-Based Integrated Framework for Network Traffic Analysis and Intrusion Detection for CPSs in IIoT

The steps of the proposed framework are shown in Algorithm 1. It provides a detailed approach to analyzing the network traffic, identifying intrusions in industrial IoT settings, and incorporating edge computing as a means of processing data in real time. It begins with the raw network traffic data collected from CPSs in the IIoT, referred to as the raw data. These data are then preprocessed through normalization, which means scaling the features in the given range between 0 and 1, making the dataset normalized. This step is important because it helps in cases where the data are going to be used repeatedly. The next phase involves network traffic analysis, in which two clustering algorithms, namely, k-means and DBSCAN, are implemented. The k-means algorithm is used for clustering data, for which minimization of the variance within clusters is the main objective. The process of clustering is achieved by assigning each data point to the correct cluster center, and the efficiency of the generated clustering is estimated through the silhouette coefficient, measuring the appropriateness of the data points belonging to a specific cluster. At the same time, DBSCAN is performed to find dense areas and assess the outliers through parameters for the size of the neighborhood and minimum points. This density-based method works in conjunction with k-means by identifying any clustering that is otherwise labeled as outliers. The silhouette score is also used to measure the level of efficiency that comes with DBSCAN clustering.

The intrusion detection process follows, utilizing three machine-learning models: k-nearest neighbors, random forest, and logistic regression. The normalized dataset divides the entire dataset into training and testing sets. K-nearest neighbors aimed to assign a class to each test instance as the majority from the nearest neighbors in a training set, random forest makes a team of decision trees; the prediction is made by a simple voting system of all the trees, while logistic regression employs a probability model for predicting the probability of intrusion using a cost function. These models present a sound way of detecting intrusions and abnormalities. To facilitate the real-time processing of data, the proposed framework incorporates edge computing, through which information is processed near the source, thus increasing efficiency. It also enhances the prompt identification and the subsequent handling of prospective threats by shifting computational tasks to the edge.

The algorithm also has decision-making based on the data collected from clustering and intrusion detection to decide the subsequent action to be taken. If there is a high probability that an anomaly or an intrusion occurred in the system, then the system can alert the administrators or even quarantine the culprits’ devices. In the case where no problems are flagged, the process proceeds without changes to the pre-existing plan. The algorithm contains a performance assessment. The performances of the clustering models are measured with the silhouette score, and the performances of the intrusion detection models are evaluated by the precision, recall, F1 score, and AUC. The confusion matrices are evaluated to estimate the accuracy of each model to distinguish between normal and attack traffic and to confirm the effectiveness of the proposed framework to detect potential threats in industrial IoT environments.
**Algorithm 1:** Edge-Computing-Based Integrated Framework for Network Traffic Analysis and Intrusion Detection for CPSs in IIoTBegin**Input:** $D_{RAW}$: Raw network data from the CPS in IIoT**Output:** Cluster-based IDS**Procedure:** Edge-Computing-Based Integrated Framework for Network Traffic Analysis and Intrusion Detection for CPSs in IIoT**Data Collection:**Collect raw network traffic data from the CPS in IIoT $$D_{RAW}=\{x_{1},\;x_{2},\;\ldots \;,\;x_{n}\}$$**Data Preprocessing:**Preprocess the raw data $D_{RAW}$ using normalization to scale features within [0, 1]$$D_{norm}=x_{i}^{norm}=\frac{x_{i}-\min\left(x\right)}{\max\left(x\right)-\min\left(x\right)},\;\forall \;x_{i}\;\in D_{RAW}$$**Network Traffic Analysis:**Apply k-means clustering to $D_{norm}$◦Minimize intra-cluster variance$${arg}_{c}^{min}\;\sum_{i=1}^{k}\sum_{x_{j}\in C_{i}}{\left|\left|x_{j}-\mu_{i}\right|\right|}^{2}$$where $\mu_{i}$ is the centroid of cluster $C_{i}$ and $C_{i}$ represents the set of points in cluster i◦Assign each data point $x_{j}$ to the closest cluster$$Cluster\;\left(x_{j}\right)={arg}_{c}^{min}\;\left|\left|x_{j}-\mu_{i}\right|\right|$$◦Computing the silhouette score $S_{K-Means}$$$S_{K-Means}=\frac{b\left(x\right)-a\left(x\right)}{\max\left(a(x\right),\;b(x))}$$where a (x) is the mean intra-cluster distance, and b (x) is the mean nearest-cluster distance
Apply DBSCAN clustering to $D_{norm}$**for** each point ($x_{j}$)   {   classify core points if it has at least MinPts neighbors within E   classify border point if it is within E of a core point but has fewer than MinPts neighbors   Outlier otherwise   }**end for**   ◦Computing the silhouette score $S_{DBSCAN}$**Intrusion Detection using Machine-Learning Models:**Let $D_{Train}$ and $D_{Test}$ represent training and testing dataset derived from $D_{norm}$Apply k-nearest neighbors◦For each test instance $x_{test}$, find k-nearest neighbors in the training set$$Neighbors\;\left(x_{test}\right)={arg}_{k}^{min}\;\left|\left|x_{test}-x_{train}\right|\right|$$◦Classify $x_{test}$ based on majority of its neighborsApply random forest
◦Construct T decision trees, each trained on random subset of $D_{Train}$◦For a test instance $x_{test}$, predict the class $y_{test}$ by averaging the predictions of all trees$$y_{test}=mode\;(\left\{h_{t}\left(x_{test}\right)\right|t=1,\;2,\;\ldots \;,\;T\})$$Apply logistic regression
◦Train LR model by fitting the parameters θ to minimize the log-loss$$\theta *={arg}_{k}^{min}\;(-\frac{1}{m}\;\sum_{i=1}^{m}[y_{i}\log\left(h_{\theta }\left(x_{i}\right)\right)+\left(1-y_{i}\right)\log(1-h_{\theta }\left(x_{i}\right))]\;)\;$$◦where $h_{\theta }\left(x\right)$ is the logistic function: $h_{\theta }\left(x\right)=\frac{1}{1+e^{-\theta^{T}x}}$Compute accuracy (A), precision (P), recall (R), and F1 score.$$A=\frac{TP+TN}{TP+TN+FP+FN\;}$$$$P=\frac{TP}{TP+FP\;}$$$$R=\frac{TP}{TP+FN\;}$$$$F1-Score=\frac{2(P\times R)}{P+R\;}$$**Edge Computing Integration and Data-Driven Decision Making:**Let $T_{Edge}$ represent the time taken for edge-based data processing and $T_{Cloud}$ represent the time for cloud-based processing$$T_{Edge}\ll T_{Cloud}$$Let A represent the set of actions triggered in response to detected intrusions$$A=\left\{\begin{array}{c} Alert\;if\;P_{anomaly}\left(x\right)>T\;\\ Isolate\;Device\;if\;P_{attack}\left(x\right)>T_{attack}\;\\ Normal\;Operation\;Otherwise\; \end{array}\right.$$where T and $T_{attack}$ are thresholds for anomaly detection and attack classificationend

## 4. Performance Evaluation

We perform simulations and evaluate the performance of the proposed framework with respect to the cluster-based network analysis and intrusion detection system.

### 4.1. Evaluation Metrics

We evaluated the performance of the methods and models implemented in the proposed framework using the silhouette score, accuracy, precision, recall, F1 score, receiver operating characteristics (ROC) curve, and precision–recall (PR) curve [45]. We calculated the accuracy, precision, recall, and F1 score based on the following terms:

- True Positive (TP): the number of correctly identified positive instances;
- True Negative (TN): the number of correctly identified negative instances;
- False Positive (FP): the number of incorrectly identified positive instances;
- False Negative (FN): the number of incorrectly identified negative instances.

The accuracy, precision, recall, and F1 scores are calculated based on the following equations [46,47]:

(1) $$A=\frac{TP+TN}{TP+TN+FP+FN}$$

(2) $$P=\frac{TP}{TP+FP}$$

(3) $$R=\frac{TP}{TP+FN}$$

(4) $$F1=\frac{2(P\times R)}{P+R}$$

The ROC and PR curves have also been used during evaluation. The ROC curve is a graphical representation of the TP rate against the FP rate at various threshold levels. The PR curve is a graphical representation of the trade-off between the precision and recall for different classification thresholds. A higher area under the curve (AUC) indicates a better performance for the model.

### 4.2. Dataset

We have utilized the dataset called NF-ToN-IoT-V2 [48], which is publicly available on the Kaggle platform. This dataset is obtained from the NFV2-collection developed by the University of Queensland to remove issues of interoperability in network security datasets for scalability. The dataset is an integrated dataset that strives to emulate the real IIoT scenario and contains information about IoT sensors, OS, and network traffic. It is labeled for several cybersecurity incidents, such as distributed denial of service (DDoS) and ransomware, among others. This is composed of normal traffic and attack traffic, which makes it suitable for use when developing machine-learning models for intrusion detection and any other security-related concerns. For this purpose, in this study, we employed this dataset for network traffic anomaly detection, where the labeled traffic data can be employed to categorize the anomalous patterns.

In the context of the proposed framework, the NF-ToN-IoT-V2 dataset is a better option for evaluating the effectiveness of network traffic analysis and intrusion detection models with our proposed framework. This dataset is specifically created to simulate realistic IIoT environments that were generated by different IoT sensors, the operating system, and network communications. It is normal and malicious traffic that encompasses many cybersecurity threats such as DDoS, ransomware, and brute force attacks. The dataset is scalable and has a rich feature set to capture the dynamic network conditions and, therefore, can be used to evaluate the robustness of intrusion detection mechanisms in IIoT scenarios. Since our frameworks include clustering methods (k-means and DBSCAN) and supervised learning methods (KNN, RF, and LR) for intrusion detection, our dataset should include as many of the different behaviors of IIoT traffic in terms of network load variation and anomaly patterns. For this, the real-world-inspired traffic flows are incorporated in the dataset, which leads to the adequate training and evaluation of the model with the labeled instances of cyber threats available in the dataset.

### 4.3. Experimental Design

The experiments were performed by implementing two clustering methods, k-means [49,50] and DBSCAN [51], and three ML models, k-nearest neighbors [34,35,36], random forest [34,37,38], and logistic regression [34,36,39,40]. The dataset has been divided into two parts: the training set and the test set. The training set comprised 70% of the total records in the dataset. The test set comprised 30% of the total number of records. All experiments are implemented in Python 3.11.8 in a GPU-based environment. Predefined ML packages and libraries including Pandas 1.24.0, Numpy 1.5.3, Seaborn 0.11.2, Sklearn 1.1.3, LabelEncoder (from Scikit-learn), OneHoTencoding (from Scikit-learn), and Matplotlib 3.6.3 have been used.

### 4.4. Results and Discussion

#### 4.4.1. Network Traffic Analysis

A network traffic pattern analysis was conducted using the k-means clustering approach, in which the “IN_BYTES” and “OUT_BYTES” parameters were used. We then grouped the network traffic into five separate categories based on the 5000 data points collected. K-means clustering was selected as it can be used for the analysis of datasets with several variables to discover underlying trends and, thus, enable us to investigate possible traffic abnormalities or different behavior patterns in the network. The distribution of instances across the five clusters is shown in Table 2.

**Figure 3:**
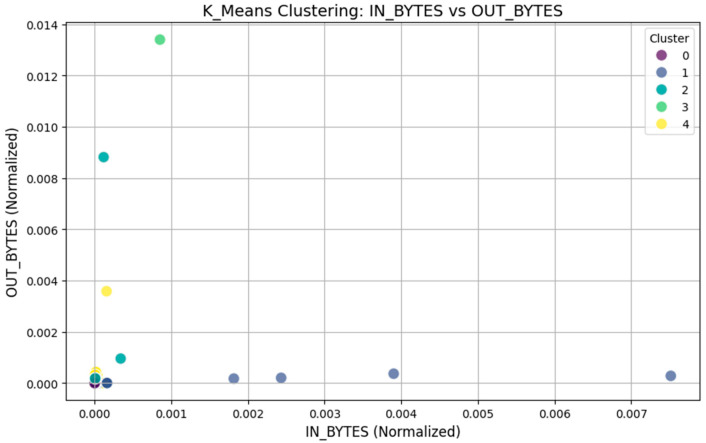
K-means clustering: IN_BYTES Vs. OUT_BYTES.

To determine the performance of the clustering, the silhouette score was used as a measure of clustering quality in order to know how well the clusters were separated. From this, a score of 0.612 was obtained, which is good for clustering quality since the clusters are well-separated. Figure 3 shows a graphical representation of the clustering solution based on the analysis of the “IN_BYTES” and “OUT_BYTES” instances normalized for analysis. Different colors have been used to represent various clusters. The x-axis plot shows that some of the clusters are spread more widely, which may imply different traffic densities or traffic patterns, while other clusters are found to be as near to zero as possible, which could imply normal traffic or a lack of traffic in the network. This analysis is important in identifying patterns that may be indicative of anomalous traffic behavior and, hence, improve security and optimization in industrial IoT systems.

Apart from k-means clustering, we used DBSCAN clustering to analyze the network traffic and search for regular/abnormal traffic patterns. DBSCAN is especially useful when the clusters are irregular in shape and size and contain noise. For the current analysis, the DBSCAN algorithm was used under parameters that include epsilon (eps) of 0.1 and min_samples of 5. When analyzing the results of resource utilization, the algorithm found several clusters, each characterizing a group of similar traffic patterns in the network. Entropy for the DBSCAN clustering was also calculated and we obtained a silhouette score of 0.473. It is lower than the score obtained here by k-means; DBSCAN is more robust against noise, and its results may shed a lot of light on isolating traffic, which could be a sign of possible anomalies or security threats.

**Figure 4:**
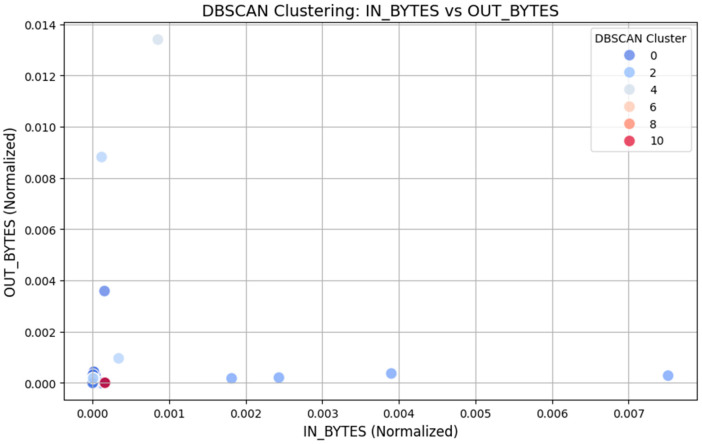
DBSCAN clustering: IN_BYTES Vs. OUT_BYTES.

The outcomes of DBSCAN clustering are shown in Figure 4, with IN_BYTES normalized in the x-axis and OUT_BYTES normalized in the y-axis. The clusters can be uniquely colored, with different shades belonging to different groups of clusters. DBSCAN also focuses on how the data are distributed, and can easily spot regions with fewer database points, which is potentially an area of noise or outliers.

The network traffic analysis with k-means and DBSCAN clustering algorithms is quite different in their approach. The most popular and fast-calculation algorithm, K-Means, divides the set into five clusters according to the “IN_BYTES” and “OUT_BYTES” attributes. The clustering yielded a silhouette coefficient of 0.612, which shows that the clustering resulted in a high separation of network traffic patterns. However, k-means is sensitive to the choice of k, also assumes clusters are spherical, and fails to work with noisy clusters or clusters of an irregular shape; thus, its ability to identify anomalies or outliers in datasets, especially those that are complex, may not be as effective. DBSCAN clustering, on the other hand, is density-based, efficient for discovering clusters of any shape, and effective in the presence of noise. It also did a better job in identifying the low-density areas that might be representative of abnormal or infrequent traffic patterns. Even though it is more flexible than k-means, DBSCAN achieved a silhouette coefficient of 0.473 because it is designed to identify noise in the data rather than creating compact and clearly separated clusters. That makes it especially important for detecting possible security breaches or other suspicious activities in network communication, where outliers and low-density data are important to find.

The silhouette scores quantify the level of clustering quality; however, the use of k-means and DBSCAN is not based on these scores alone but on their strengths in dealing with different IIoT network traffic patterns. Our framework is based on the use of the NF-ToN-IoT-V2 dataset, which contains heterogeneous traffic with normal operations and different attack scenarios. However, such variability suggests that k-means is useful for segmenting structured patterns efficiently, whereas DBSCAN is good at detecting anomalies and noise. In our proposed framework, we employ both of them as they help in a comprehensive analysis via k-means to identify the principal traffic clusters and DBSCAN for outliers and irregular traffic behavior. We have also investigated the stability and interpretability of clusters by considering intra-cluster variance and the effectiveness of detecting the anomalies.

#### 4.4.2. Intrusion Detection System

We implemented and evaluated three ML models, KNN, RF, and LR. All these models have expertise in identifying network intrusions in the industrial IoT networks. KNN is a distance-based classifier, and it is good at capturing the local structures and finding the outliers in the dataset. RF, as one of the popular ensemble methods, provides a high efficiency in dealing with large datasets with the high dimensionality of features and does not allow overfitting. LR, which is a probabilistic model, is particularly useful for binary classification such as intrusion detection and can explain the relative association between features. Comparing the results of the use of these models, we identify the best strategy for detecting intrusions in real time within cyber–physical systems. The results of the IDS models, i.e., KNN, RF, and LR, are shown in Table 3 and demonstrate varying levels of performance across multiple metrics.

In the benign class, the precision of the RF was 1.00, indicating that all instances that the RF model classified as benign are indeed benign. KNN was also good with a precision value of 0.99, while the LR had a lower value of 0.96, which means that the model predicted a few false positives. On the same note, the recall of RF and KNN stood at 0.98 and that of LR was 0.89. From this, it can be inferred that LR failed to identify more benign cases, lumping them together with the attacks. RF emerged the best with an F1 score of 0.99, followed by KNN. However, the F1 score that was calculated for LR was 0.92, which indicates that the model’s recall is worse than its precision. Based on accuracy, as seen above, RF and KNN produced almost similar results with an accuracy of 0.99 for benign traffic, while LR, though equally accurate, had a slightly lower value of 0.97.

In the case of the attack class, KNN and LR are both valued at 1.00 for the precision, which means that the classifying models did not misclassify any attack instances, and all the instances are correctly labeled as attacks. The RF model gave a slightly lower accuracy of 0.99, which also proved good enough. For recall, all models achieved a perfect recall of 1.00 on attack detection except for the LR model, which achieved 0.99. This means that, although LR was active, it did not miss very many attacks, which is not true according to the results. The F1 scores also supported this trend, with both KNN and RF models having the same F1 score of 1.00, but with F1 metrics being the harmonic means of precision and recall. LR has a slightly lesser F1 measure of 0.98, but it is also significantly good.

When comparing the results of the models on macro averages across both benign and attack classes, all models were good, with RF having the highest performance with scores of 1.00 in precision, recall, and F1. KNN, with an average of 0.99, showed a good trade-off between both true benign and true malicious activities. LR slightly performed worse with 0.97 precision, 0.94 recall, and 0.95 F1 score and was slightly less accurate and able to detect the benign class. In the same manner as the accuracy averages, which also take into account the number of instances in each class, RF and KKN achieved very high scores of 0.99 in all the metrics, implying a robustness in both benign and attack classes. LR had a slightly lower weighted average of 0.97 because it lowered its detection performance for benign traffic.

Random forest (RF) showed the highest accuracy for the total score and the highest specific, non-specific, and overall accuracy of the attack and benign classes. This is the reason why the performance of RF is better since it can cope with the complicated data patterns with an appropriate degree of predictability. The KNN model also provided good results, even better than the previous models, especially for attacks where all the metrics were scored 100%. But it had a slightly lower recall in the case of benign instances as compared to RF, which means that there may be a small number of benign events being classified as an attack. Although LR was good at recognizing attacks, it had deficiencies in identifying benign traffic, as depicted by its low recall and F1 scores for benign samples. Finally, RF was identified as being the most accurate model for intrusion detection, providing good accuracy across the two classes. KNN was also very efficient in this case, especially in the identification of attacks. While LR has been valuable for the purposes of this paper, some problems have been identified with its handling of benign traffic and the overall possibility of more false positives. According to the above findings, RF seems to be the most appropriate for real-time IDSs in an edge-computing-based cyber–physical system.

**Figure 5:**
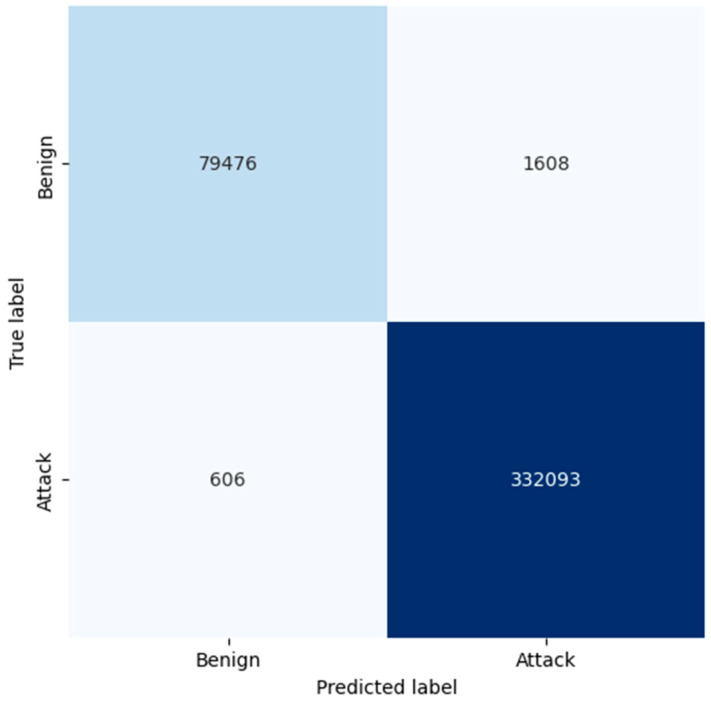
Confusion matrix generated by KNN.

**Figure 6:**
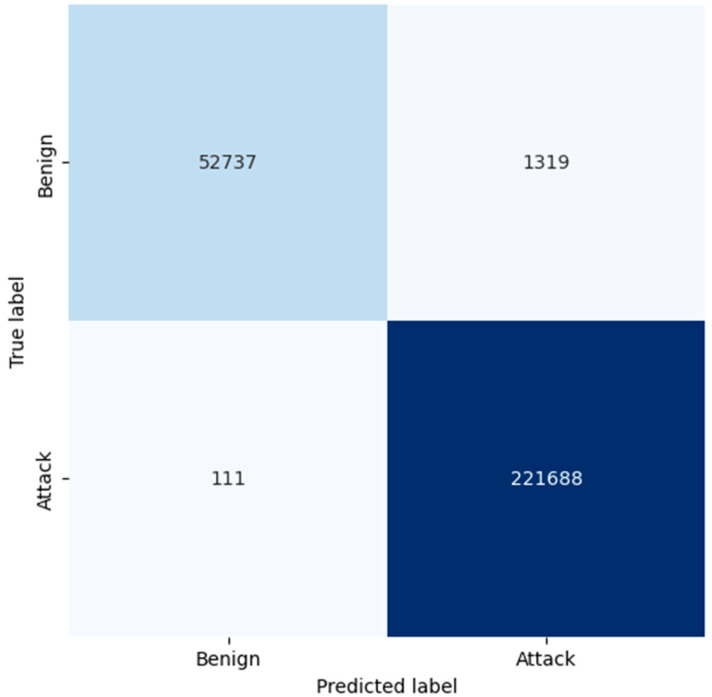
Confusion matrix generated by RF.

**Figure 7:**
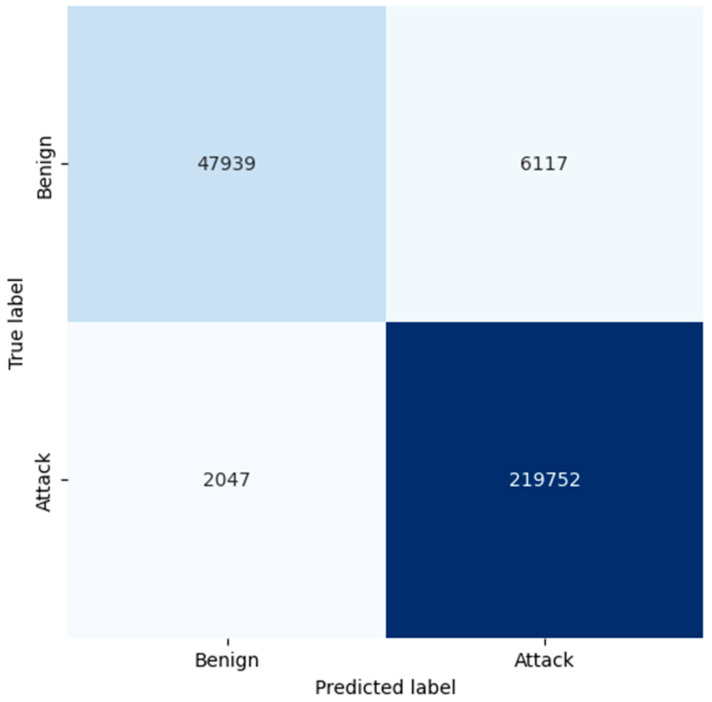
Confusion matrix generated by LR.

The confusion matrices of KNN, RF, and LR, as shown in Figure 5, Figure 6 and Figure 7, present the analysis results of the classification models on the identification of benign and malicious traffic. For KNN, the confusion matrix depicts that the model has successfully classified benign traffic with 79,476 TP and 1608 FP. This leads to an accuracy of 98% and a specificity of 99% for benign instances, which show that KNN is accurate enough in identifying benign traffic. However, for the attack detection, the model has a moderate number of false negatives (606), which means that some of the attacks were not detected at all, which may be an issue in critical applications where all possible threats have to be flagged.

The confusion matrix of RF proves that the model has good accuracy in general. For benign traffic, the true positives are at 52,737, while the false positives are at 1319, resulting in a high level of precision of 0.98 for benign classification through RF. Even more critical, in the case of attack detection, RF has a small number of false negatives equal to 111, so the recall of the algorithm is 1.00. As demonstrated, this makes RF more reliable in detecting attacks with low false negative rates and in accurately categorizing legitimate traffic. From the confusion matrix, the LR model identifies the attacks reasonably well, while four times more false positives are recorded from the benign traffic when compared to both KNN and RF. The model found 47,939 true positives for benign traffic and 6117 false positives; this means it requires more time to analyze, thus giving a lower precision of 0.92. Moreover, it made 2047 false negatives to attacks, and this affected its recall in providing complete details of all possible threats. The larger ratio of false positive cases makes one believe that LR might be classifying benign traffic as malicious more often than it should, which might be disastrous, especially in environments where minimizing false alarms is important.

**Figure 8:**
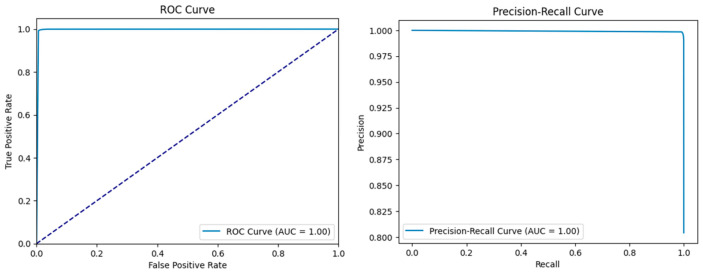
ROC and precision–recall curves for KNN.

**Figure 9:**
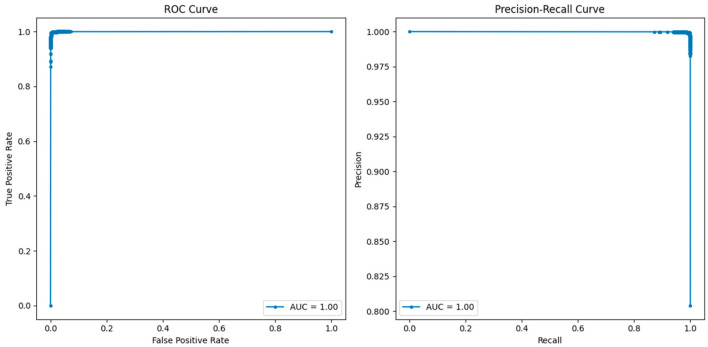
ROC and precision–recall curves for RF.

**Figure 10:**
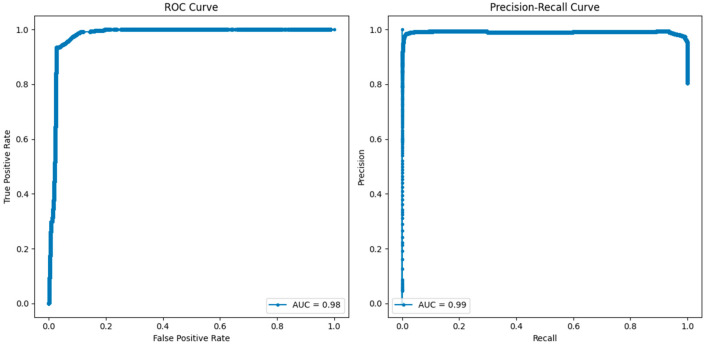
ROC and precision–recall curves for LR.

The comparative analysis of the ROC and precision–recall curves, as shown in Figure 8, Figure 9 and Figure 10, gives a detailed insight into the results of the KNN, RF, and LR models. In the case of KNN, the optimum results can be observed from the ROC as well as the precision–recall curves, where the obtained values of both curves are equal to one. The ROC curve of true positive and false positive rates gives us an insight that KNN has a sensitivity and specificity of 100% at different thresholds, meaning that KNN does not produce any false positives or false negatives in the classification of benign and malicious traffic. The precision–recall curve also depicts the value of 1.00, which also confirms that KNN has the perfect value of precision and recall. This seems to imply that KNN is very efficient in differentiating between benign and attack instances with no degradation in its capability. In terms of both the ROC and precision–recall curve, RF also showed the best result of one. The results of the ROC suggest that, for RF, there is a perfect distinction between benign traffic and attack traffic and that, even at a high TPR, the FP rate is very low.

The AUC of the precision–recall curve also stands at 1, which confirms that RF has the potential of achieving both high precision and recall, which means that all instances, whether they are benign or an attack, will be accurately classified. The above evaluation of RF in these measures shows that it is a reliable intrusion detection model for detecting intrusion and assessing the intrusion tolerance of complex systems. Logistic regression, though slightly lower in terms of accuracy compared to the best-performing KNN and RF algorithms, is also quite good. The area under the curve in the case of LR is 0.98, which means that the test is very sensitive and specific but not 100%. The precision–recall curve shows 0.99 and depicts the aspect of precision and recall but a slightly lower score compared to KNN and RF algorithms. This suggests that, even though LR is efficient in identifying the attacks and correctly categorizing the benign traffic, there is a slightly lower performance compared to the other models.

The novelty of our framework lies in its multicriteria integration of network traffic analysis, clustering, machine-learning-based intrusion detection, and edge computing for real-time security enhancement. We integrate k-means and DBSCAN for network traffic clustering, allowing anomaly detection in unlabeled data. We then employ KNN, RF, and LR for supervised intrusion detection, enhancing the ability to classify cyber threats with high accuracy. Instead of relying solely on cloud-based analysis, we provide a concept of task distribution to edge nodes (e.g., routers, fog nodes, and gateways) for real-time security monitoring. The traffic analysis through clustering using k-means and DBSCAN successfully revealed outstanding traffic clusters and anomalies in the network. K-means clustering was seen to be very effective for clustering the dataset into easily distinguished clusters, which helped in the profiling of traffic behavior well. The concept of the silhouette score was used here to justify the acceptable number of clusters, which is 7, with a silhouette score of 0.612, which gave a reasonable measure/degree of cluster separation to identify the traffic patterns with a reasonable level of accuracy. However, DBSCAN clustering, which allowed noise and strange forms of clusters, showed regions of low density that may indicate unusual or malicious activity. Even though the silhouette score was significantly lower with DBSCAN at 0.473, its capabilities of finding outliers and noise would be beneficial to the k-means clustering in offering a robust analysis of the network traffic-related patterns.

The subsequent intrusion detection analysis using KNN, RF, and LR models presented here proved the effectiveness of these approaches for detecting benign and malicious traffic. KNN and RF were almost flawless with all the metrics, ROC and precision–recall, showing how efficient these algorithms are in classifying traffic and identifying potential threats. As it unveiled, KNN presented an adequate figure both in terms of precision and recall; therefore, the use of this model in real-time intrusion detection was suitable. Between the four models tested, RF came out as the most accurate in identifying all the malicious activities due to its ability to accommodate complicated data patterns and its low number of false negatives. Compared to that of LR, the performance of other algorithms, although slightly worse, was still satisfactory, especially in terms of attack detection with high accuracy. Another key area for integration is that edge computing becomes critical for managing and analyzing network traffic data in a non-stop manner and in real time. The system is able to stay adaptive to the high volumes of data produced in industrial IoT settings because edge computing is used to manage the data loads away from central servers.

## 5. Conclusions

This research work introduces an edge-computing-centered integrated solution for network traffic analysis and intrusion detection that improve CSP security in IIoT. The k-means and DBSCAN algorithms are used in the framework to analyze traffic patterns and classify them as normal; if any traffic pattern deviates from the normal traffic patterns, then the traffic pattern is classified as a possible security threat. The clusters generated by k-means clustering have a silhouette score of 0.612, thus proving that this method can effectively classify data into proper clusters, while the DBSCAN clustering algorithm has a silhouette score of 0.473, thereby showing the ability of the algorithm to detect noise and outliers in network traffic. These clustering methods are thus used as a starting point for subsequent security analysis to improve traffic behavior understanding. In intrusion detection, the model was used, and the application of machine-learning models was carried out and closely tested. KNN and RF models were also at the top of the ranking, receiving 1.00 for both the ROC and precision–recall scores. Just slightly less accurate was logistic regression, which still had an ROC of 0.98 and a precision–recall score of 0.99. These results are a clear indication of the viability of the machine-learning technique in detecting intrusions with few false positives and false negatives, and, thus, increase the reliability of the detector. The confusion matrices support this and indicate, for example, that RF specifically was significantly better at correctly categorizing benign and malicious traffic. The use of edge computing integration is an important element of the framework. By carrying out most of the processing on the edge, the system reduces the latency, hence making it possible to provide real-time decisions and freeing the cloud center resources. This architecture is appropriate for use in industrial IoT scenarios since a high response time to threats is required to sustain operation. Edge computing also brings scalability since the system can easily expand as the IoT networks grow without much negative impact on performance, let alone security issues.

In future, we will focus on refining ML models by integrating time-series analysis and advanced communication protocols. We aim to explore hybrid algorithms to improve risk assessment and anomaly identification, especially against more sophisticated attack types in CPSs. We also plan to implement fog-computing-based fuzzy logic systems to optimize 5G communication technology performance in IIoT.

## Figures and Tables

**Table 1:** A comparison of the proposed framework with previous studies.

Reference	Focused Area	Machine Learning Techniques	Features	Dataset	Key Contributions
[13]	Anomaly detection in CPS for Supply Chain 4.0	PCA-BSO feature selection + ML models	Network traffic anomalies	Real-world traffic data from a semiconductor factory	PCA-BSO used to select significant features, improving classification accuracy
[16]	Security issues in cyber–physical systems (CPSs)	Review-based work	CPS security threats and attack types	Review-based work	Categorizes CPS security threats and describes philosophical challenges
[27]	Edge computing integration in CPS	Review-based work	QoS optimization in CPS	Review-based work	Edge–cloud computing and QoS challenges in CPSs
[41]	Machine-learning-based cybersecurity analysis	Network IDS and Host IDS	Attack pattern analysis, adversarial ML vulnerabilities	KDDCup99, NSL-KDD, UNSW-NB15, WSN-DS, CICIDS 2017	Explores robustness of IDSs against adversarial ML attacks
[42]	IDS in Edge Intelligence for Smart Cities	Linear Correlation Feature Selection, INTERACT, MLP	Feature extraction and selection for attack prediction	KDDCup99	Hybrid feature selection and ML for improved intrusion detection
Proposed Framework	Network traffic analysis and intrusion detection in IIoT CPS using edge computing	k-means, DBSCAN, KNN, RF, LR	Network traffic behavior, anomaly detection, supervised learning for intrusion detection	NF-ToN-IoT-V2 (real-world industrial IoT dataset)	Integrates clustering and ML models with edge computing for real-time and scalable intrusion detection

**Table 2:** Distribution of instances across the five clusters.

Cluster	Number of Instances
Cluster 4	2089
Cluster 0	1808
Cluster 1	624
Cluster 2	393
Cluster 3	86

**Table 3:** Comparative analysis of accuracy, precision, recall, and F1 score.

Class	Precision			Recall			F1 Score			Accuracy		
	KNN	RF	LR	KNN	RF	LR	KNN	RF	LR	KNN	RF	LR
Benign (0)	0.99	1.00	0.96	0.98	0.98	0.89	0.99	0.99	0.92	0.99	0.99	0.97
Attack (1)	1.00	0.99	0.97	1.00	1.00	0.99	1.00	1.00	0.98			
Macro average	0.99	1.00	0.97	0.99	0.99	0.94	0.99	0.99	0.95			
Weighted average	0.99	0.99	0.97	0.99	0.99	0.97	0.99	0.99	0.97			

## Data Availability

The dataset supporting the findings of this study is publicly available on a Kaggle website with the title NF-ToN-IoT-V2 [48].
